# Efficacy of a Dietary Supplement Extracted from Persimmon (*Diospyros kaki* L.f.) in Overweight Healthy Adults: A Randomized, Double-Blind, Controlled Clinical Trial

**DOI:** 10.3390/foods13244072

**Published:** 2024-12-17

**Authors:** Silvia Pérez-Piñero, Juan Carlos Muñoz-Carrillo, Jon Echepare-Taberna, Cristina Herrera-Fernández, Macarena Muñoz-Cámara, Vicente Ávila-Gandía, Francisco Javier López-Román

**Affiliations:** 1 Faculty of Medicine, UCAM Universidad Católica San Antonio de Murcia, Carretera de Guadalupe s/n, 30107 Murcia, Spain; sperez2@ucam.edu (S.P.-P.); jcmunoz@ucam.edu (J.C.M.-C.); cherrera@ucam.edu (C.H.-F.); mmunoz5@ucam.edu (M.M.-C.); vavila@ucam.edu (V.Á.-G.); jlroman@ucam.edu (F.J.L.-R.); 2 Primary Care Research Group, Biomedical Research Institute of Murcia (IMIB-Arrixaca), 30120 Murcia, Spain

**Keywords:** obesity, overweight, persimmon, *Diospyros kaki*, dietary supplement, clinical trial

## Abstract

A single-center, randomized, double-blind, and placebo-controlled clinical trial assessed the efficacy in improving body composition and in weight management of a dietary supplement consisting of 400 mg of a standardized extract of the persimmon fruit (*Diospyros kaki* L.f.) in adult subjects with a BMI between 25 and 34.99 kg/m^2^ administered for 120 consecutive days. In total, 36 participants were assigned to the placebo group and 35 to the experimental group (registered at ClinicalTrials.gov (NCT05750342)). Primary analysis focused on overweight subjects (placebo, *n* = 26; experimental, *n* = 23). In this group, fat mass expressed in kg and percentage evaluated by both dual-energy X-ray absorptiometry (DEXA) and bioelectrical impedance analysis (BIA) decreased significantly (between-group differences *p* < 0.001) in those receiving the persimmon extract as compared with the placebo. No significant reduction in lean mass was observed, suggesting that the muscle mass was maintained during fat loss. The use of the investigational product improved classic anthropometric parameters to a statistically significantly greater extent than the placebo, including body weight, BMI, and waist and abdominal circumference (*p* < 0.001), in the overweight group. In the overall population, similar improvements were observed, with significant between-group differences (*p* < 0.001) in fat mass reduction and improvements in body composition. Changes in the biochemical lipidic, glycemic, and anti-inflammatory profile were not found, except for between-group significant differences (*p* < 0.001) in decreases in tumor necrosis factor-alpha (TNFα) and increases in total antioxidant capacity (TAC) in favor of the experimental condition. There was a significant increase in fecal fat excretion in the experimental group at the end of the study in subjects with low fecal fat (9%) at baseline. Consumption of the investigational product vs. placebo improved the quality of life, with significantly greater scores in the total score and the mental health component of the SF-12 questionnaire. The persimmon extract was safe and well tolerated.

## 1. Introduction

Overweight and obesity are among the main lifestyle illnesses leading to major public health concerns for their health risks and represent a rapidly spreading global problem [1]. Overweight, defined as a body mass index (BMI) over 25 kg/m^2^, and obesity, defined as a BMI over 30 kg/m^2^, have become increasingly prevalent worldwide. According to data of the World Health Organization (WHO), the percentage of overweight adults has risen dramatically, contributing to a growing risk of developing obesity-related comorbidities [2]. The World Obesity Federation in the World Obesity Atlas 2024 reported that global levels of high BMI are projected to increase from 46% of adults in 2025 to over 54% by 2035 with nearly 3.3 billion adults [3].

Addressing overweight, as an early intervention target, is crucial in mitigating the progression towards obesity and the related health risks. Although obesity is a complex and multifactorial condition, its prevention and management begin with tackling overweight, which is largely preventable through lifestyle modifications, dietary changes and supplementation. The importance of weight management lies in preventing obesity, and also in the reduction in the risks of insulin resistance, hypertension, and hyperlipidemia and other non-communicable diseases [4].

Persimmon (*Diospyros kaki* L.f.) is an important fruit in Asia that has expanded rapidly in Europe and the Mediterranean basin. Persimmon is a carbohydrate and fiber-rich fruit that is low in fat and calories, providing a high variety of vitamins and antioxidants, which helps to maintain satiety during weight loss dietary practices. Numerous bioactive components, including polyphenols (especially tannins), procyanidins, carotenoids, flavonoids, triterpenoids, amino acids, fatty acids, vitamins (A and C), and mineral elements, have been associated with the health benefits of persimmon, including anti-inflammatory, antioxidant, antimicrobial, antidiabetic, hypolipemic, and anticancer activities [5,6,7,8,9,10]. In relation to the lipid-lowering effect, persimmon leaves have been reported to improve lipid profiles and suppress body weight gain [11]. In an experimental mice model of obesity, persimmon tannins showed an inhibitory effect on pancreatic lipase, suggesting a reduction in fat absorption via inhibition of pancreatic lipase [12]. In high-fat diet-fed rats, persimmon leaf extract lowered body fat weight and improved plasma and hepatic liver profiles [13]. In another study in mice fed with a high-fat diet, fermented persimmon extract (FPE) supplementation led to an approximate 15% reduction in body weight, reduced abdominal and liver fat, and reduced serum levels of triglycerides, total cholesterol, and glucose, suggesting that gallic acid, a potent major bioactive component in FPE, exerts potent effects via AMP-activated protein kinase [14].

Despite evidence provided by these studies, the potential benefits of persimmon extract in clinical settings, particularly for overweight individuals, have not previously been assessed. Therefore, the objective of this clinical trial was to determine the efficacy of a dietary supplement consisting of a standardized persimmon extract in improving body composition, particularly in overweight adults. By focusing on overweight subjects, we aim to provide insights into early-stage interventions that may prevent the transition to obesity and its associated health risks.

## 2. Materials and Methods

### 2.1. Study Design and Participants

This was a prospective, randomized, double-blind, placebo-controlled clinical trial with two parallel arms carried out at the Health Science Department of Universidad Católica San Antonio de Murcia (UCAM), in Murcia, Spain. The study began on 2 November 2023 and finished on 18 March 2024. The primary objective of the study was to evaluate the efficacy of daily consumption of a dietary supplement with a standardized persimmon extract for 120 days in overweight subjects (BMI 25–29.99 kg/m^2^). Secondary analyses included the efficacy in subjects with class 1 obesity (BMI 30–34.99 kg/m^2^), as well as assessment of anthropometric data; lipid, anti-inflammatory, and glycemic profiles; plasma total antioxidant capacity; fecal fat excretion; health-related quality of life; and safety.

Eligible participants were males and females aged between 18 and 65 years, with a BMI > 25 and <35 kg/m^2^ and were able to understand and fulfill the requirements of the study. Special focus was placed on recruiting overweight subjects, while class 1 obese participants were also included for secondary analyses. Subjects were excluded in the presence of at least one of the following criteria: current treatments that may affect body weight; acute disease; history or presence of pulmonary, hepatic, renal, hematological, gastrointestinal, endocrine, immunological, dermatological, urological, neurological, psychiatric or cardiovascular disease, or active malignancy; major surgery within the past 3 months; subjects that quit smoking in the last 6 months or intended to quit during the study; food allergy or eat disorders; concomitant participation in another study that included blood sampling or a dietary intervention; pregnant or lactating women; and refusal to provide informed consent.

The study protocol was approved by the Research Ethics Committee of Universidad Católica San Antonio de Murcia (UCAM) (code CE122202, approval date December 22, 2022) and was registered at ClinicalTrials.gov (NCT05750342). Protection of personal data according to Organic Law No. 3/2018 of 5 December 2018 was ensured. All participants were fully informed of the purpose of the study and signed the written informed consent form.

### 2.2. Randomization and Intervention

Participants who met the inclusion criteria were randomized (1:1) using a simple randomization procedure to the experimental group (persimmon extract supplement) or the control group (placebo supplement) using the Epidat 4.2 software program. An independent researcher was responsible for the randomization process.

The tested dietary ingredient was an extract of persimmon fruit, *Diospyros kaki* L.f. (perFix^™^, Euromed S.A., Mollet del Vallès, Barcelona, Spain). The product consisted of a greenish brown powder with a moisture content lower than 6% and the composition of which included no less than 68% of *Diospyros kaki* L.f. and no more than 30% maltodextrin, and 2% anhydrous colloidal silica as excipients were added to prevent agglomeration. The methodology for obtaining the fruit extract from locally sourced persimmon fruits was based on an eco-friendly ultrapure continuous water extraction method and refinement by a proprietary tangential flow filtration process (Pure-Hydro Process^®^). The correct management of the raw material and an accurate extraction method provided a total concentration of condensed tannins higher than 5%. This amount of active ingredients was determined by a high-performance liquid chromatography (HPLC) method that uses UV spectroscopy as a detection system. The nutritional value of the persimmon fruit extract is shown in Table S1 of the Supplementary Materials.

The placebo capsules contained magnesium stearate and microcrystalline cellulose. The study products were delivered as identically appearing capsules and had the same organoleptic characteristics. Subjects assigned to the experimental group were recommended to take two capsules daily each containing 200 mg of the standardized persimmon extract, (one about 30 min before breakfast, and the other about 30 min before the main meal) for 120 consecutive days (4 months). Subjects in the control group followed the same regimen. All participants received instructions regarding the importance of not introducing changes in their standard dietary patterns, particularly avoiding foods that contain flavonoids (chocolate, nuts, coffee, tea, etc.), as well as to communicate to the principal investigator the introduction of any new pharmacological medication during the study. Compliance was defined as the consumption of at least 80% of the study product (capsules taken divided by total number of capsules [*n* = 240] × 100, so that only 48 capsules corresponding to 24 days out of 120 days could be left).

### 2.3. Study Procedures

Patients attended a baseline visit (day 0, visit 1) and two visits during the course of the study at day 60 (mid-study, visit 2) and at day 120 (end of study, visit 3). Approximately within ±10 days prior to visit 1, participants were evaluated at the study center in order to assess fulfilment of the inclusion criteria, to record their complete medical history, to obtain the signed informed consent forms, and to perform randomization of subjects to the study groups.

At visit 1 (baseline), demographic and anthropometric characteristics were registered and participants received their corresponding study product for the following treatment period of 60 days. Study procedures included assessment of body composition by dual-energy X-ray absorptiometry (DEXA) and bioelectrical impedance analysis (BIA), health-related quality of life (HRQL), fecal fat excretion, level of physical activity by actigraphy, plasma total antioxidant capacity, lipid, anti-inflammatory and glycemic profiles, a dietary survey, and routine safety testing.

At visit 2 (mid-study), the same analyses as those of the baseline, except for fecal fat excretion and plasma total antioxidant capacity, and lipid, anti-inflammatory and glycemic profiles, were performed. In addition, the study product for the next 60 days of treatment was provided and the capsules returned were counted. Adverse events (AEs) were recorded.

At visit 3 (the end of the study), the same procedures as those of baseline were performed. AEs were registered and the capsules returned were counted.

### 2.4. Study Variables

General characteristics of patients included demographics, anthropometric variables, and blood pressure (BP) (systolic [SBP] and diastolic [DBP]). Anthropometric measurements included weight, height, BMI, and waist, hip and abdominal circumference in both men and women). Body composition was assessed by DEXA using the Norland XR-46 bone densitometry system, measuring total fat mass, percentage of total fat mass, total lean mass, trunk fat mass, percentage of trunk fat mass, and trunk lean mass. Body composition was also measured by BIA using the whole-body BIA analyzer Tanita BC-420MA (Tanita BC-420MA, Tanita Corp., Tokyo, Japan), assessing fat mass, percentage of fat mass, lean mass, and muscle mass.

Biochemical analyses were performed using 12 h fasting blood samples obtained by venipuncture and included a lipid profile (serum levels of total cholesterol, triglycerides, high-density lipoprotein (HDL) cholesterol, and low-density lipoprotein (LDL) cholesterol); anti-inflammatory markers (serum levels of C-reactive protein [CRP], interleukin (IL) 6 [IL-6], and tumor necrosis factor alpha [TNFα]); glycemic profile (serum levels of glucose, glycated hemoglobin, and baseline insulin); and plasma total antioxidant capacity (TAC). Fecal fat analysis was performed using an infrared spectroscopic method [15].

The HRQL was measured using the Impact of Weight on Quality of Life-Lite Clinical Trials version (IWQOL-Lite-CT), which is a validated, 20-item patient-reported outcome (PRO) measure designed to assess the impact of changes in weight on patients’ physical and psychosocial functioning [16]. Each item employs a 5-point graded response scale (from never to always, or from not at all true to completely true) yielding a total score and scores for the physical domain, physical function, and psychosocial domain. Scores range from 0 to 100, with higher scores reflecting better levels of functioning. A Spanish validated version of the 12-item Short Form Survey (SF-12) questionnaire was also used to assess HRQL [17]. The SF-12 includes 12 questions that measure 8 health domains to assess overall quality of life (total score) and physical and mental health components. Scores range from 0 to 100, with higher scores indicating better HRQL.

The potential bias in the study was controlled by measuring the level of physical activity and conducting a dietary survey. The level of physical activity was evaluated using a wrist-worn accelerometer (ActiGraph wGT3X-BT accelerometer, ActiGraph, Pensacola, FL, USA) during 3 days of the week and 1 day of the weekend, with results expressed as metabolic equivalents (METs). The 24 h recall method was used for the dietary survey. Data were recorded over a 7-day period and analyzed using Dietsource^®^ v1.2 software. The percentages of proteins, lipids, and carbohydrates consumed were analyzed.

Safety data included BP recording and laboratory analyses. Standard hematological (hemogram) and biochemical parameters (renal and liver function tests) were measured.

### 2.5. Statistical Analysis

The analysis was based on the per-protocol (PP) data set corresponding to those participants who met the inclusion criteria and completed the study at 120 days. Analyses included the comparison of the experimental group vs. placebo in the whole study population and in the subset of overweight subjects (BMI ≥ 25 and <29.99 kg/m^2^). Categorical variables are expressed as frequencies and percentages, and quantitative variables as mean ± standard deviation (SD) and range. The chi-square test was used for the comparison of quantitative variables and Student’s *t* test for the comparison of quantitative variables. Changes in variables in the study groups over the course of the study were analyzed with the analysis of variance (ANOVA) for repeated measures with two study factors: within subject factor (baseline, 60 days [mid-visit], and 120 days [end of study]) and between-subject factors (intervention: active product and placebo) for paired data. Post hoc analyses were performed with Bonferroni’s correction or Tukey’s procedure. Statistical significance was set at *p* < 0.05. Data were analyzed with the Statistical Package for the Social Sciences (SPSS) version 25.0 (IBM Corp., Armonk, NY, USA).

## 3. Results

### 3.1. General Characteristics of the Study Population

**Figure 1:**
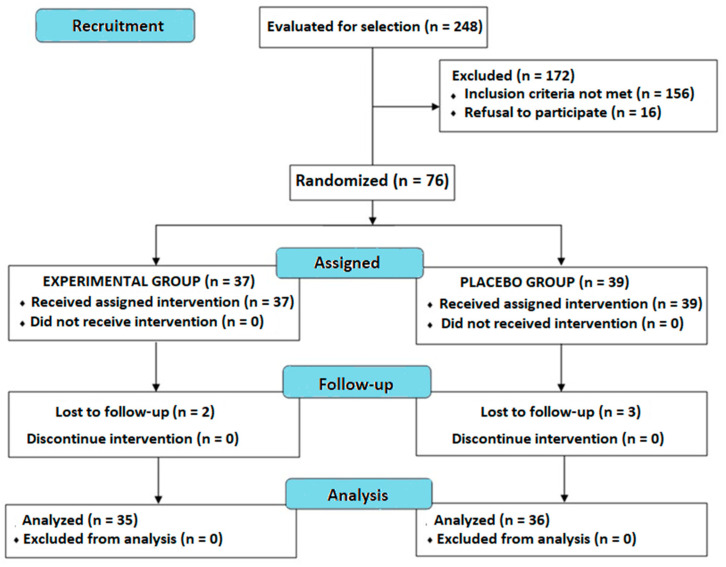
Flow chart of the study population.

A total of 248 candidates were recruited, but 156 did not meet the inclusion criteria and were excluded. Moreover, 16 candidates refused to take part in the study. Therefore, the initial study population consisted of 76 subjects, 37 of which were randomized to the experimental group and the remaining 39 were assigned to the placebo group. However, five subjects (experimental group, *n* = 2; placebo group, *n* = 3) failed to attend the study visits and were excluded from the analysis. A total of 71 subjects (experimental group, *n* = 35; placebo group, *n* = 36) completed the study. Figure 1 shows the flow chart of the study subjects.

There were 42 men and 29 women, with a mean age of 31.5 ± 13.5 years, body weight of 85.8 ± 12.8 kg, and BMI of 29.1 ± 3.1 kg/m^2^. A total of 49 subjects (69.0%) were overweight with a mean BMI of 27.2 ± 1.4 kg/m^2^ and the remaining 22 had class 1 obesity with a mean BMI of 33.1 ± 2.1 kg/m^2^. The baseline characteristics of participants are shown in Table 1.

### 3.2. Body Composition Evaluated by DEXA

In overweight subjects (Table 2), the experimental group showed significant reductions in fat mass (kg and percentage), trunk fat mass, and percentage of trunk fat mass from baseline to the end of the study (*p* < 0.001). Between-group comparisons also revealed statistically significant differences in favor of the experimental group (*p* < 0.001) with significant reductions already observed at the mid-study visit. No significant changes were observed in lean mass in either the experimental or placebo groups.

In the overall population, including both overweight and class 1 obesity subjects, the experimental group demonstrated similar significant reductions in fat mass, percentage of fat mass, and trunk fat mass (*p* < 0.001) compared to the placebo group. However, in subjects assigned to the placebo group, fat mass showed a significant increase during the study period (from 28.3 ± 7.1 kg at baseline to 29.4 ± 7.3 kg at the end of the study, *p* < 0.001). Increases in the percentage of fat mass were also significant (from 33.8 ± 8.2% to 34.8 ± 8.2%, *p* = 0.012). Within-group differences in the remaining variables were not significant. A summary of the results obtained in the overall study population, including both overweight and class 1 obesity subjects, is shown in Table 3.

### 3.3. Body Composition Evaluated by BIA

In overweight subjects, the experimental group showed a significant reduction in fat mass and percentage of fat mass (*p* < 0.001) compared to the placebo group, while no significant changes were observed in lean mass or muscle mass. These findings were consistent with the results from the DEXA analysis. Between-group differences in fat mass were significant (*p* < 0.001), with the experimental group showing greater improvements. Fat mass decreased significantly in subjects assigned to the experimental group, both in the overall study population and in the subset of patients with overweight, as compared with the subjects that took the placebo. Within-group differences in these variables were also statistically significant in the experimental group. In the overall population, similar trends were observed, with significant reductions in fat mass and percentage of fat mass in the experimental group compared to the placebo (Table 4). Changes in lean mass and muscle mass were not observed in any of the study groups.

### 3.4. Anthropometric Variables

The results of anthropometric variables are shown in Table 5. In overweight subjects, the experimental group demonstrated significant reductions in body weight, BMI, waist circumference, and abdominal circumference compared to both the baseline and placebo (*p* < 0.001). In the overall study population, there was a significant decrease in body weight in the experimental group as compared with the placebo (between-group differences *p* = 0.001). Weight also decreased significantly during the course of the study in the experimental group (mean 2.2 kg). The same findings were found in the analysis of overweight subjects. However, in subjects treated with the placebo, there was an increase in weight, particularly between the mid-study and the final visits (mean 0.7 kg, *p* < 0.05). In relation to BMI, the experimental group showed significant decreases both in the overall study population and in overweight subjects, with statistically significant between-group differences (*p* < 0.001). Similar findings were observed for changes in waist circumference and abdominal circumference.

Other anthropometric variables showing statistically significant reductions in the experimental group as compared to the placebo were the waist and the abdominal circumferences in men, and the hip circumference in all study subjects and in women (Table S2 of the Supplementary Materials).

### 3.5. Lipid, Anti-Inflammatory, and Glycemic Profiles

The parameters of the lipid profile, including total cholesterol, triglycerides, HDL cholesterol, and LDL cholesterol, did not show significant differences when the values obtained at baseline and at the end of the study were compared both in the overall study subjects and in overweight subjects. Similar findings were found for the glycemic profile without significant changes in serum levels of glucose, glycated hemoglobin, and insulin. In relation to serum anti-inflammatory markers, serum levels of CRP and Il-6 did not show significant changes, but serum levels of TNFα decreased significantly both in the placebo group (from 10.33 ± 3.27 to 8.78 ± 4.06 pg/mL, *p* < 0.001) and the experimental group (from 10.33 ± 1.45 to 6.77 ± 1.73 pg/mL, *p* < 0.001), but the decrease rate was higher in the experimental group (between-group differences *p* = 0.001). The results of all these variables are shown in Table S3 of the Supplementary Materials.

Plasma TAC levels increased significantly in both study groups, i.e., from 379.5 ± 95.4 to 414.1 ± 106.8 mmol Fe^2+^ in the placebo group (*p* < 0.050) and from 373.1 ± 113.5 to 479.9 ± 96.9 mmol Fe^2+^ in the experimental group (*p* < 0.001), but differences were higher in the experimental group (between-group differences *p* = 0.008).

### 3.6. Fecal Fat Excretion

The percentage of fat excretion was not modified during the study, and between-group differences were not statistically significant (*p* = 0.407). However, statistically significant between-group differences (*p* = 0.040) were found in a subanalysis of subjects with a low percentage of fecal fat excretion (<9%). This analysis showed that in subjects assigned to the experimental group, fecal fat excretion increased from 5.51 ± 2.48% at baseline to 9.86 ± 9.83% at the final visit (*p* = 0.008), whereas in the placebo group, there was practically no increase (from 4.22 ± 2.28% to 4.06 ± 3.21%, *p* = 0.912).

### 3.7. Quality of Life

The analysis of HRQL using the IWQOL-Lite-CT instrument did not show between-group significant differences in any domains of the questionnaire, although baseline scores were higher than 70, indicating that study subjects already had a good quality of life before starting dietary supplementation. There were within-group statistically significant improvements in the total score and the psychosocial domain score in the two study groups over the course of the study. Detailed results are shown in Table S4 of the Supplementary Materials. However, the analysis of HRQL using the SF-12 questionnaire showed statistically significant improvements in the total score and the mental health component in subjects assigned to the experimental group as compared with the placebo group (Table 6). Differences in the physical health component between the study groups were not found, but differences between the baseline and final visits increased significantly among subjects in the experimental group only (from 78.1 ± 15.8 to 82.7 ± 13.7; *p* < 0.034).

### 3.8. Level of Physical Activity and Dietary Survey

The study groups did not modify any variables related to the control of bias after dietary supplementation with the active product or placebo for 120 days. In relation to the levels of physical activity, METs showed a mean value of 1.72 ± 0.22 and 1.68 ± 0.20 at baseline in the placebo and the experimental group, respectively, which were almost the same at the end of the study (1.70 ± 0.24 and 1.65 ± 0.25, respectively).

There was a loss of calories during the study period, both in the placebo and in the experimental group, with within-group statistically significant differences. The loss of fat followed a similar decreasing trend, but between-group differences were not statistically significant for any of the nutritional parameters. The results of the dietary survey are shown in Table S5 of the Supplementary Materials.

### 3.9. Compliance and Safety

Consumption of at least 80% of the study product was confirmed in all study subjects, with a maximum number of 32 capsules returned by one participant. No abnormal changes in SBP, DBP and heart rate were observed during the study period. Safety of the investigational product was confirmed by laboratory analyses with results of all blood and biochemical tests within normal limits.

## 4. Discussion

This clinical trial conducted in overweight/class 1 obesity adult subjects shows that the consumption of a dietary supplement consisting of persimmon fruit extract for 120 consecutive days was associated with statistically significant improvements in body composition (particularly in overweight subjects) measured by DEXA, BIA, and standard anthropometric parameters as compared to subjects assigned to the placebo group.

The decreases in fat mass and trunk fat mass, evaluated by DEXA, with statistically significant differences as compared with the placebo group were remarkable. It should be noted that decreases in fat mass were already recorded at the mid-study visit at about 60 days after starting the use of the investigational product. Dietary consumption of supplements with a standardized persimmon fruit extract decreased the amount and percentage of fat mass to a significantly greater extent in comparison with the consumption of placebo. Interestingly, when body composition was evaluated by BIA, fat mass expressed both in kg and as a percentage decreased in the experimental group, with statistically significant differences versus the placebo group. The improvements in body composition with reduced fat mass were associated with a significant increase in fecal fat excretion, although this finding was limited to those subjects with a low percentage (9%) of fat excretion at baseline.

These favorable changes in body composition documented by DEXA and BIA in subjects assigned the experimental group were also recorded in the evaluation of classic anthropometric indicators, including reductions in body weight, BMI, and waist and abdominal circumference in overweight subjects and in all subjects with significant differences as compared with the placebo. Moreover, significant decreases in the experimental group were recorded in waist circumference in men, hip circumference in all subjects and in women, and abdominal circumference in men, reflecting improvements in body contouring. These results suggest the importance of weight management as well as the preservation of lean mass since no significant losses in muscle mass were reported.

Persimmon fruit is a good source of nutritional antioxidants, carotenoids, and polyphenols, especially proanthocyanidins (tannins), all of which have been involved in the health and medicinal benefits of persimmon, including cardiovascular disease, anti-diabetic effect, anticancer properties, improvement of the lipid profile, anti-allergic, and reduction in skin ageing [18]. Owing to rich phytochemistry, persimmon is considered effective for the pharmacological application of its functional ingredients that may help against hyperlipidemia and hyperglycemia [19]. In relation to the modulation of the lipid metabolism and the anti-obesity properties of persimmon, different experimental studies have demonstrated a hypocholesterolemic effect [20] that appears to be related to the tannin content, bile acid-binding capacity [21,22,23], the inhibition of adipogenesis and downregulation of fatty acid synthesis with an increase in transcription factors associated with fatty acid oxidation [24], the reduction in fat absorption through the inhibition of pancreatic lipase [9,25], the decrease in the protein levels of fatty acid synthase, stimulation of AMP-activated protein kinase (AMPK) phosphorylation, and down-regulation of genes involved in lipogenesis [26]. Also, suppression of inflammatory cytokines such as TNFα, CRP, and the protein level of nuclear factor-kappa B (NF-*k*B) in the liver have been shown in rats fed a high-fat diet [26].

However, despite these data in experimental animals, there are a few investigations of the weight management properties of persimmon fruits in humans. In a randomized, placebo-controlled trial, the effect of persimmon-based cookie bars containing different doses of tannin-rich fiber (low-dose 3 g, high-dose 5 g) was evaluated in 40 subjects with increased cholesterol levels [27]. Participants ingested tannin-rich fiber bars 3 times daily before the meals for 12 weeks. Cholesterol levels decreased significantly at 6 and 12 weeks in the experimental groups, with decreases in LDL cholesterol in the high-dose group, whereas no changes were found in the placebo group. The authors concluded that tannin-rich fiber from young persimmon fruits is a useful food material for treating hypercholesterolemia [27]. In our study, changes in the lipid and glycemic profiles were not found. It may be tentatively argued that an effect of persimmon extract could have been observed in subjects with hypercholesterolemia and/or hypertriglyceridemia or hyperglycemia at baseline, but laboratory parameters were within normal limits in all participants. A longer duration of persimmon supplementation could also influence the detection of possible changes in the lipid profile. The interventional product, however, was able to significantly reduce the levels of anti-inflammatory markers, particularly TNFα with statistically significant differences as compared with the placebo. Decreases in CRP and IL-6 were also statistically significant in the experimental group over the course of the study. In agreement with the antioxidant properties of persimmon, statistically significant higher increases in plasma TAC levels over the study period were found in the experimental group.

Dietary supplementation with the investigational product also improved HRQL assessed with the SF-12 questionnaire, in which scores in all domains increased significantly during the study, whereas in the placebo group, no significant increases were recorded. Moreover, between-group significant differences for the total score and the mental health component score were found, indicating better psychological well-being and suggesting an improvement in mood and emotional stability. In the IWQOL-Lite-CT questionnaire, differences between the study groups were not observed, although there were significant improvements in the total score and the psychosocial domain during the study, both in the experimental and the placebo groups. There was also a trend for greater improvements in the experimental group. It is likely that the reduced sample size may account for the lack of differences between the experimental and the placebo groups found in the IWQOL-Lite-CT questionnaire.

Finally, modification of bias control-related variables (level of physical activity and dietary survey) was not found. The investigational product was safe, well tolerated, and showed a high compliance rate. Previous studies support the selection of the 200 mg dose of the standardized persimmon extract. In an experimental study in a model of obese and non-obese mice, in which the anti-obesity effects of the 100 and 200 mg doses of young persimmon fruit aqueous extract were compared, reduction in triglyceride and total cholesterol concentrations occurred in a dose-dependent manner [24]. In a randomized, double-blind, placebo-controlled trial that aimed to assess the hypocholesterolemic effects of cookie bars containing 0 g (placebo group), 3 g (low-dose group), or 5 g (high-dose group) of tannin-rich persimmon fiber 3 times daily before meals for 12 weeks, body weight and body fat percentage were not affected throughout the study period, but both the 300 and 500 mg doses reduced plasma cholesterol levels [27].

The present findings should be interpreted considering the limitations of the study, especially the reduced number of participants in each study arm, which affects the generalizability of results, and the duration of the dietary intervention of 120 days. The inclusion of a well-established fat-lowering agent (reducing fat absorption by inhibiting pancreatic lipase as a similar mechanism to that proposed for persimmon tannins) as a positive control could add evidence of the validity of the present findings by providing an additional standard for the effects of persimmon extract. However, the strengths of the study are the randomized controlled design and the assessment of a large number of variables. These variables included the effect of the intervention on body composition, anthropometric measurements, and biochemical analyses (objective data) and HRQL (subjective data). To our knowledge, no previous study using a persimmon-based dietary supplement in the overweight/obesity setting has been carried out.

## 5. Conclusions

In overweight/class 1 obesity healthy adult subjects, the administration of a dietary supplement providing a daily amount of 400 mg of a natural extract of persimmon (*Diospyros kaki* L.f.) over 120 days significantly improved fat mass-related variables, while maintaining muscle mass. The primary analysis focused on overweight participants, where the beneficial effects on body composition were particularly notable. No significant changes in lean mass were observed, indicating that fat loss occurred without compromising muscle mass, which is important for maintaining healthy body composition. The weight reduction observed was accompanied by significant reductions in waist and abdominal circumference. The properties of persimmon extract to support weight management, fat loss and body composition improvement, with evidence of safety and no adverse events, in humans is a relevant finding that should be evaluated in further studies with a larger sample size in order to validate the present results and enhance robustness of our data.

## Figures and Tables

**Table 1:** Baseline data of the study population.

Variables	Total Subjects	Placebo Group	Experimental Group
Men/women	42/29	22/14	20/15
Age, years	31.5 ± 13.5	31.7 ± 13.6	31.6 ± 13.6
Weight, kg	85.8 ± 12.8	85.7 ± 12.8	85.6 ± 12.8
BMI, kg/m^2^	29.1 ± 3.1	29.1 ± 3.1	29.1 ± 3.1
Overweight subjects, *n* = 49	27.2 ± 1.4	27.0 ± 1.3	27.5 ± 1.5
Class 1 obesity subjects, *n* = 22	33.1 ± 2.1	33.3 ± 2.3	32.9 ± 2.1
Systolic BP, mmHg	123.4 ± 15.9	123.5 ± 16.2	123.3 ± 16.0
Diastolic BP, mmHg	78.4 ± 9.4	78.5 ± 9.6	78.3 ± 9.5

BMI: body mass index; BP: blood pressure.

**Table 2:** Changes in body composition assessed by DEXA in overweight subjects.

Variables and Study Groups	Visit 1 Baseline	Visit 2 Mid-Study (60 days)	Visit 3 Final (120 days)	Within-Group Differences Visits 1 vs. 3 *p* Value	Between-Group Differences *p* Value
Fat mass, kg					
Placebo (*n* = 26)	25.1 ± 4.7	25.6 ± 5.3	26.2 ± 5.3	0.014	0.001
Experimental (*n* = 23)	26.4 ± 5.6	25.5 ± 5.2 *	24.3 ± 5.2	<0.001	
Fat mass, %					
Placebo (*n* = 26)	31.7 ± 7.5	32.2 ± 7.4	32.8 ± 7.8	0.028	0.001
Experimental (*n* = 23)	33.1 ± 7.8	32.3 ± 7.2 *	31.2 ± 7.5	<0.001	
Lean mass, kg					
Placebo (*n* =26)	55.3 ± 11.5	55.0 ± 10.9	54.8 ± 11.5	0.442	0.292
Experimental (*n* = 23)	54.5 ± 11.7	54.6 ± 11.4	54.7 ± 11.5	1.0	
Trunk fat mass, kg					
Placebo (*n* = 26)	12.5 ± 2.9	12.4 ± 2.9	12.2 ± 3.0	1.0	0.091
Experimental (*n* = 23)	14.0 ± 3.3	13.5 ± 3.2 *	12.9 ± 3.0	<0.006	
Trunk fat mass, %					
Placebo (*n* = 26)	15.9 ± 4.3	15.7 ± 4.1	15.3 ± 4.2	0.370	0.192
Experimental (*n* = 23)	17.4 ± 4.0	16.8 ± 3.8 *	16.1 ± 3.6	<0.003	
Trunk lean mass, kg					
Placebo (*n* = 26)	25.2 ± 5.4	25.2 ± 5.0	25.1 ± 5.0	1.0	0.101
Experimental (*n* = 23)	24.5 ± 5.0	24.5 ± 4.9	24.8 ± 5.0	0.143	

* Statistical significance (*p* < 0.05) in the evolution of the variable in the intermediate measurement compared to baseline. DEXA: dual-energy X-ray absorptiometry. Overweight defined as BMI between 25 and 29.99 kg/m^2^.

**Table 3:** Changes in body composition assessed by DEXA in the overall study population.

Variables and Study Groups	Visit 1 Baseline	Visit 2 Mid-Study (60 days)	Visit 3 Final (120 days)	Within-Group Differences Visits 1 vs. 3 *p* Value	Between-Group Differences *p* Value
Fat mass, kg					
Placebo (*n* = 36)	28.3 ± 7.1	28.9 ± 7.4	29.4 ± 7.3	<0.001	<0.001
Experimental (*n* = 35)	30.1 ± 8.1	29.3 ± 7.8 *	28.1 ± 7.8	<0.001	
Fat mass, %					
Placebo (*n* = 36)	33.8 ± 8.2	34.3 ± 8.0	34.8 ± 8.2	0.012	<0.001
Experimental (*n* = 35)	34.7 ± 7.7	34.1 ± 7.3 *	33.1 ± 7.5	<0.001	
Lean mass, kg					
Placebo (*n* =36)	56.2 ± 11.8	55.6 ± 10.9	55.8 ± 11.7	0.720	0.677
Experimental (*n* = 35)	56.9 ± 11.2	56.7 ± 10.6	56.8 ± 10.6	1.0	
Trunk fat mass, kg					
Placebo (*n* = 36)	14.4 ± 4.2	14.4 ± 4.3	14.2 ± 4.4	1.0	0.010
Experimental (*n* = 35)	16.5 ± 5.1	16.0 ± 5.2 *	15.4 ± 5.0	<0.001	
Trunk fat mass, %					
Placebo (*n* = 36)	17.2 ± 4.6	17.1 ± 4.5	16.8 ± 4.6	0.304	0.050
Experimental (*n* = 35)	18.8 ± 4.3	18.3 ± 4.4 *	17.6 ± 4.2	<0.001	
Trunk lean mass, kg					
Placebo (*n* = 36)	25.6 ± 5.5	25.7 ± 4.9	25.5 ± 5.0	1.0	0.207
Experimental (*n* = 35)	25.6 ± 4.9	25.7 ± 4.8	25.8 ± 4.6	0.415	

* Statistical significance (*p* < 0.05) in the evolution of the variable in the intermediate measurement compared to baseline. DEXA: dual-energy X-ray absorptiometry.

**Table 4:** Body composition in all study subjects and in overweight subjects evaluated by BIA.

Variables and Study Groups	Visit 1 Baseline	Visit 2 Mid-Study (60 days)	Visit 3 Final (120 days)	Within-Group Differences Visits 1 vs. 3 *p* Value	Between-Group Differences *p* Value
**Fat mass, kg**					
All subjects					
Placebo (*n* =36)	24.6 ± 7.0	24.9 ± 7.2	25.2 ± 7.2	0.210	<0.001
Experimental (*n* = 35)	27.6 ± 8.4	27.4 ± 8.2	26.4 ± 8.3	<0.001	
Overweight subjects					
Placebo (*n* = 26)	21.2 ± 4.1	21.6 ± 4.6	21.9 ± 4.5	0.482	<0.001
Experimental (*n* = 23)	23.5 ± 5.2	23.3 ± 5.0	22.1 ± 5.1	0.013	
**Fat mass, %**					
All subjects					
Placebo (*n* =36)	29.3 ± 7.7	29.6 ± 7.7	29.7 ± 7.6	0.666	0.014
Experimental (*n* = 35)	31.6 ± 7.8	31.8 ± 7.8	31.0 ± 8.3	0.207	
Overweight subjects					
Placebo (*n* = 26)	26.9 ± 6.5	27.2 ± 6.6	27.3 ± 6.4	0.835	0.010
Experimental (*n* = 23)	29.4 ± 7.2	29.6 ± 7.2	28.5 ± 7.7	0.142	
**Lean mass, kg**					
All subjects					
Placebo (*n* =36)	59.9 ± 11.5	59.6 ± 10.9	60.0 ± 11.2	1.0	0.277
Experimental (*n* = 35)	59.0 ± 10.9	58.7 ± 11.0	58.6 ± 11.2	0.214	
Overweight subjects					
Placebo (*n* = 26)	59.1 ± 11.2	58.9 ± 10.8	59.2 ± 11.0	1.0	0.565
Experimental (*n* = 23)	57.3 ± 11.5	56.8 ± 11.5	56.9 ± 12.0	0.125	
**Muscle mass, kg**					
All subjects					
Placebo (*n* =36)	56.6 ± 11.2	56.3 ± 10.7	56.7 ± 11.0	1.0	0.581
Experimental (*n* = 35)	56.5 ± 10.5	56.0 ± 10.2	57.0 ± 10.6	0.145	
Overweight subjects					
Placebo (*n* = 26)	55.8 ± 11.1	55.6 ± 10.7	55.8 ± 10.8	1.0	0.243
Experimental (*n* = 23)	54.5 ± 10.8	54.5 ± 10.9	55.1 ± 11.0	0.079	

**Table 5:** Changes in body weight, BMI and waist and abdominal circumference in all study subjects and in those who were overweight.

Variables and Study Groups	Visit 1 Baseline	Visit 2 Mid-Study (60 days)	Visit 3 Final (120 days)	Within-Group Differences Visits 1 vs. 3 *p* Value	Between-Group Differences *p* Value
**Weight, kg**					
All subjects					
Placebo (*n* =36)	84.5 ± 12.3	84.5 ± 11.8	85.2 ± 12.4	0.192	0.001
Experimental (*n* = 35)	87.1 ± 13.2	86.0 ± 12.8 *	84.9 ± 12.6	<0.001	
Overweight subjects					
Placebo (*n* = 26)	80.4 ± 10.3	80.5 ± 10.1	81.0 ± 10.5	0.593	0.001
Experimental (*n* = 23)	80.9 ± 10.5	80.1 ± 10.5	79.0 ± 10.3	<0.003	
**BMI, kg/m^2^**					
All subjects					
Placebo (*n* =36)	28.8 ± 3.3	28.8 ± 3.2	29.0 ± 3.3	0.168	0.001
Experimental (*n* = 35)	29.3 ± 3.1	29.0 ± 3.0 *	28.6 ± 3.0	<0.001	
Overweight subjects					
Placebo (*n* = 26)	27.0 ± 1.3	27.1 ± 1.4	27.2 ± 1.4	0.613	0.001
Experimental (*n* = 23)	27.5 ± 1.5	27.2 ± 1.6	26.9 ± 1.7	<0.003	
**Waist circumference, cm**					
All subjects					
Placebo (*n* =36)	90.6 ± 8.6	90.5 ± 8.4	90.7 ± 8.9	1.0	0.015
Experimental (*n* = 35)	93.8 ± 10.9	93.5 ± 10.6	92.1 ± 10.3	<0.004	
Overweight subjects					
Placebo (*n* = 26)	87.1 ± 6.0	87.2 ± 5.8	87.1 ± 6.1	1.0	0.040
Experimental (*n* = 23)	88.2 ± 8.0	88.0 ± 7.7	86.3 ± 6.8	<0.010	
**Abdominal circumference, cm**					
All subjects					
Placebo (*n* =36)	92.1 ± 9.7	91.8 ± 10.6	92.6 ± 10.0	1.0	0.002
Experimental (*n* = 35)	95.9 ± 11.9	94.1 ± 12.5 *	93.7 ± 12.2	<0.004	
Overweight subjects					
Placebo (*n* = 26)	88.0 ± 6.5	87.2 ± 7.0	88.2 ± 7.2	1.0	0.007
Experimental (*n* = 23)	89.9 ± 8.0	87.7 ± 7.9 *	87.2 ± 7.5	<0.007	

* Statistical significance (*p* < 0.05) in the evolution of the variable in the intermediate measurement compared to baseline. Overweight is defined as BMI between 25 and 29.99 kg/m^2^.

**Table 6:** Health-related quality of life using the SF-12 questionnaire in the study population.

Domains and Study Groups	Visit 1 Baseline	Visit 2 Mid-Study (60 days)	Visit 3 Final (120 days)	Within-Group Differences Visits 1 vs. 3 *p* Value	Between-Group Differences *p* Value
Total score					
Placebo (*n* =36)	71.3 ± 14.1	72.2 ± 15.6	71.4 ± 17.2	1.0	0.028
Experimental (*n* = 35)	73.2 ± 13.7	77.4 ± 13.2 *	78.5 ± 12.8	<0.014	
Physical health component score					
Placebo (*n* =36)	79.9 ± 12.6	79.0 ± 14.0	79.5 ± 16.0	1.0	0.104
Experimental (*n* = 35)	78.1 ± 15.8	80.7 ± 14.6	82.7 ± 13.7	<0.034	
Mental health component score					
Placebo (*n* =36)	65.8 ± 17.4	66.1 ± 18.0	66.3 ± 20.1	1.0	0.045
Experimental (*n* = 35)	69.8 ± 15.8	75.1 ± 16.1 *	75.9 ± 15.9	<0.040	

* Statistical significance (*p* < 0.05) in the evolution of the variable in the intermediate measurement compared to baseline.

## Data Availability

The original contributions presented in this study are included in the article/Supplementary Materials. Further inquiries can be directed to the corresponding author.

## Supplementary Materials

The following supporting information can be downloaded at https://www.mdpi.com/article/10.3390/foods13244072/s1: Table S1: Nutritional value of the study product (per 100 g); Table S2: Anthropometric variables in the study population; Table S3: Lipid, anti-inflammatory, and glycemic profile in the study population; Table S4:Quality of life using the Impact of Weight on Quality of Life-Lite Clinical Trials version (IWQOL-Lite-CT) in the study population; Table S5: Calorie and protein, fat, and carbohydrate consumption during the study.
