# Supplementary material for: Efficacy of a Dietary Supplement Extracted from Persimmon (Diospyros kaki L.f.) in Overweight Healthy Adults: A Randomized, Double-Blind, Controlled Clinical Trial
Source: Foods. 2024 Dec 17;13(24):4072. doi: 10.3390/foods13244072 (PMC11675947; doi:10.3390/foods13244072)
Supplement: Supplementary file 1 [file foods-13-04072-s001.zip › Supplementary material Table S1.pdf]

## Supplementary material

**Table S1.** Nutritional value of the study product (per 100 g).

| Component | Value | Unit |
|---|---|---|
| Energy | 363 | Kcal |
| | 1541 | kJ |
| Total lipids | 0.7 | g |
| Total protein | 2.9 | g |
| Water | 4.1 | g |
| Carbohydrates | 84.6 | g |
| Total fiber | 3.6 | g |
| Total sugars | 60.2 | g |
| Minerals | | |
| Calcium | 57 | mg |
| Iron | 0.3 | mg |
| Potassium | 1200 | mg |
| Sodium | 33 | mg |
