# Supplementary material for: Efficacy of a Dietary Supplement Extracted from Persimmon (Diospyros kaki L.f.) in Overweight Healthy Adults: A Randomized, Double-Blind, Controlled Clinical Trial
Source: Foods. 2024 Dec 17;13(24):4072. doi: 10.3390/foods13244072 (PMC11675947; doi:10.3390/foods13244072)
Supplement: Supplementary file 1 [file foods-13-04072-s001.zip › Supplementary material Table S2.pdf]

Supplementary material

Table S2. Anthropometric variables in the study population

Variables and study subjects	Visit 1 Baseline	Visit 2 Mid-study (60 days)	Visit 3 Final (120 days)	Within-group differences *p* value	Between-group differences *p* value
**Waist circumference in men, cm**					
Placebo (n = 22)	93.6 ± 7.9	93.6 ± 8.1	93.8 ± 8.3	1.0	0.027
Experimental (n = 20)	98.3 ± 9.0	97.7 ± 8.8	96.2 ± 8.6	< 0.008	
**Hip circumference, cm**					
Placebo (n = 36)	107.9 ± 6.0	107.6 ± 6.6	107.4 ± 6.7	1.0	0.031
Experimental (n = 35)	110.5 ± 7.5	109.3 ± 6.9*	108.0 ± 7.7	< 0.001	
**Hip circumference in women, cm**					
Placebo (n = 14)	108.8 ± 7.0	109.0 ± 8.0	108.5 ± 7.9	1.0	< 0.050
Experimental (n = 15)	113.1 ± 8.8	110.8 ± 7.7*	110.1 ± 8.4	< 0.009	
**Waist-hip ratio, cm**					
All subjects					
Placebo (n = 36)	0.84 ± 0.07	0.84 ± 0.07	0.85 ± 0.07	1.0	0.778
Experimental (n = 35)	0.85 ± 0.09	0.86 ± 0.08	0.85 ± 0.08	1.0	
Overweight subjects					
Placebo (n = 26)	0.83 ± 0.06	0.83 ± 0.06	0.84 ± 0.06	1.0	0.959
Experimental (n = 23)	0.83 ± 0.09	0.83 ± 0.08	0.83 ± 0.08	1.0	
**Abdominal circumference in men, cm**					
All subjects					
Placebo (n = 22)	94.5 ± 9.4	95.1 ± 9.9	95.5 ± 9.2	0.637	0.001
Experimental (n = 20)	100.4 ± 9.6	98.0 ± 10.3*	97.1 ± 11.0	< 0.001	
Overweight subjects					
Placebo (n = 16)	90.3 ± 5.1	90.3 ± 5.0	91.3 ± 5.5	0.789	0.001
Experimental (n = 12)	94.8 ± 5.4	91.8 ± 5.4*	90.5 ± 6.5	< 0.001	

*Statistical significance ($p < 0.05$) in the evolution of the variable in the intermediate measurement compared to baseline. Overweight defined as BMI between 25 and 29.99 kg/m2.
