# Supplementary material for: Efficacy of a Dietary Supplement Extracted from Persimmon (Diospyros kaki L.f.) in Overweight Healthy Adults: A Randomized, Double-Blind, Controlled Clinical Trial
Source: Foods. 2024 Dec 17;13(24):4072. doi: 10.3390/foods13244072 (PMC11675947; doi:10.3390/foods13244072)
Supplement: Supplementary file 1 [file foods-13-04072-s001.zip › Supplementary material Table S3.pdf]

Supplementary material

Table S3. Lipid, anti-inflammatory, ang glycemic profile in the study population.

Variables and study subjects	Visit 1<br>Baseline	Visit 3<br>Final (120 days)	Between-group differences<br>*p* value
**Lipid profile**			
Total cholesterol, mg/dL			
Placebo (n = 36)	186.6 ± 41.0	189.0 ± 40.4	0.791
Experimental (n = 35)	186.6 ± 40.6	188.8 ± 41.6	
Triglycerides, mg/dL			
Placebo (n = 36)	81.8 ± 35.4	76.6 ± 35.1	0.504
Experimental (n = 35)	106.0 ± 56.0	94.8 ± 45.8	
HDL cholesterol, mg/dL			
Placebo (n = 36)	52.8 ± 13.8	51.7 ± 15.0	0.792
Experimental (n = 35)	53.7 ± 13.1	52.1 ± 13.6	
LDH cholesterol			
Placebo (n = 36)	116.2 ± 31.5	114.6 ± 29.6	0.396
Experimental (n = 35)	118.7 ± 34.7	112.1 ± 38.5	
**Anti-inflammatory biomarkers**			
C-reactive protein (CRP), mg/L			
Placebo (n = 36)	2.10 ± 1.89	3.33 ± 3.17	0.566
Experimental (n = 35)	1.06 ± 1.72	3.74 ± 2.77	
Interleukin-6 (IL-6), pg/mL			
Placebo (n = 36)	3.34 ± 2.13	2.95 ± 3.04	0.226
Experimental (n = 35)	3.54 ± 1.58	2.65 ± 1.96	
Tumor necrosis factor alpha (TNFα), pg/mL			
Placebo (n = 36)	10.33 ± 3.26	8.78 ± 4.06	0.001
Experimental (n = 35)	10.03 ± 1.45	6.77 ± 1.73	
**Glycemic profile**			
Fasting blood glucose, mg/dL			
Placebo (n = 36)	87.28 ± 9.71	85.90 ± 7.00	0.541
Experimental (n = 35)	89.99 ± 17.11	86.33 ± 13.0	
Glycated hemoglobin (HbA1), %			
Placebo (n = 36)	5.39 ± 0.45	5.17 ± 0.31	0.345
Experimental (n = 35)	5.47 ± 0.58	5.16 ± 0.53	
Fasting insulin, IU/mL			
Placebo (n = 36)	13.0 ± 5.3	13.7 ± 7.4	0.911
Experimental (n = 35)	15.9 ± 14.3	16.3 ± 12.6	

Normal ranges: total cholesterol < 200 mg/dL; triglycerides < 150 mg/dL; high-density lipoprotein (HDL) cholesterol: ≥ 60 mg/dL (desirable) < 40 mg/dL (at risk); low-density lipoprotein (LDL) cholesterol: 100-129 mg/dL; CRP: < 3 mg/L; IL-6: 7 pg/mL (upper limit); TNFα: 1-30 pg/mL; glucose: < 100 mg/dL; HbA1: < 5.7%; insulin: 5-25 IU/mL.
