# Supplementary material for: Efficacy of a Dietary Supplement Extracted from Persimmon (Diospyros kaki L.f.) in Overweight Healthy Adults: A Randomized, Double-Blind, Controlled Clinical Trial
Source: Foods. 2024 Dec 17;13(24):4072. doi: 10.3390/foods13244072 (PMC11675947; doi:10.3390/foods13244072)
Supplement: Supplementary file 1 [file foods-13-04072-s001.zip › Supplementary material Table S4.pdf]

Supplementary material

Table S4. Quality of life using the Impact of Weight on Quality of Life-Lite Clinical Trials version (IWQOL-Lite-CT) in the study population

Domains and study subjects	Visit 1 Baseline	Visit 2 Mid-study (60 days)	Visit 3 Final (120 days)	Within-group differences *p* value	Between-group differences *p* value
**Total score**					
Placebo (n = 22)	73.5 ± 15.2	76.5 ± 16.4	76.9 ± 17.3	< 0.017	0.861
Experimental (n = 20)	73.2 ± 17.8	76.4 ± 16.5	77.5 ± 16.9	< 0.002	
**Physical domain score**					
Placebo (n = 36)	75.3 ± 16.3	76.3 ± 16.7	77.4 ± 17.9	0.883	0.890
Experimental (n = 35)	71.4 ± 20.1	73.6 ± 18.4	74.5 ± 19.9	0.380	
**Physical function score**					
Placebo (n = 14)	76.5 ± 18.1	76.4 ± 18.4	77.9 ± 20.4	1.0	0.916
Experimental (n = 15)	75.3 ± 18.5	75.1 ± 19.7	75.7 ± 21.9	1.0	
**Psychosocial domain score**					
Placebo (n = 36)	72.2 ± 18.4	76.5 ± 18.3*	76.6 ± 18.7	< 0.004	0.986
Experimental (n = 35)	74.0 ± 18.9	77.9 ± 17.1*	78.0 ± 17.7	< 0.009	

*Statistical significance (p < 0.05) in the evolution of the variable in the intermediate measurement compared to baseline.
