# Supplementary material for: Efficacy of a Dietary Supplement Extracted from Persimmon (Diospyros kaki L.f.) in Overweight Healthy Adults: A Randomized, Double-Blind, Controlled Clinical Trial
Source: Foods. 2024 Dec 17;13(24):4072. doi: 10.3390/foods13244072 (PMC11675947; doi:10.3390/foods13244072)
Supplement: Supplementary file 1 [file foods-13-04072-s001.zip › Supplementary material Table S5.pdf]

Supplementary material

**Table S5.** Calories and protein, fat, and carbohydrate consumption during the study

| Variables and study groups | Visit 1 Baseline | Visit 2 Mid-study (60 days) | Visit 3 Final (120 days) | Within-group differences visits 1 vs. 3 *p* value | Between-group differences *p* value |
|---|---|---|---|---|---|
| **Calories, Kcal** | | | | | |
| Placebo (n =36) | 2037.0 ± 621.8 | 1960.4 ± 635.2 | 1780.2 ± 696.1 | < 0.004 | 0.554 |
| Experimental (n = 35) | 2055.6 ± 528.9 | 1876.4 ± 527.1* | 1793.2 ± 564.7 | < 0.004 | |
| **Proteins, %** | | | | | |
| Placebo (n =36) | 18.8 ± 4.0 | 19.3 ± 3.8 | 20.0 ± 4.5 | 0.106 | 0.966 |
| Experimental (n = 35) | 17.3 ± 3.1 | 17.8 ± 3.2 | 18.5 ± 3.6 | 0.098 | |
| **Fat, %** | | | | | |
| Placebo (n =36) | 41.4 ± 8.0 | 40.4 ± 8.4 | 38.2 ± 10.2 | < 0.018 | 0.797 |
| Experimental (n = 35) | 43.0 ± 5.4 | 40.7 ± 8.2 | 38.9 ± 6.2 | < 0.003 | |
| **Carbohydrates, %** | | | | | |
| Placebo (n =36) | 41.3 ± 7.5 | 42.3 ± 7.8 | 43.8 ± 9.1 | 0.090 | 0.930 |
| Experimental (n = 35) | 40.3 ± 6.6 | 41.0 ± 6.0 | 43.2 ± 6.6 | < 0.045 | |

*Statistical significance ($p < 0.05$) in the evolution of the variable in the intermediate measurement compared to baseline.
